# Human RECQ1 Is a DNA Damage Responsive Protein Required for Genotoxic Stress Resistance and Suppression of Sister Chromatid Exchanges

**DOI:** 10.1371/journal.pone.0001297

**Published:** 2007-12-12

**Authors:** Sudha Sharma, Robert M. Brosh

**Affiliations:** Laboratory of Molecular Gerontology, Department of Health and Human Services, National Institute on Aging, National Institutes of Health, Baltimore, Maryland, United States of America; University of Massachusetts Medical School, United States of America

## Abstract

**Background:**

DNA helicases are ubiquitous enzymes that unwind DNA in an ATP-dependent and directionally specific manner. Unwinding of double-stranded DNA is essential for the processes of DNA repair, recombination, transcription, and DNA replication. Five human DNA helicases sharing sequence similarity with the *E. coli* RecQ helicase have been identified. Three of the human RecQ helicases are implicated in hereditary diseases (Bloom syndrome, Werner syndrome, and Rothmund-Thomson syndrome) which display clinical symptoms of premature aging and cancer. RECQ1 helicase is the most highly expressed of the human RecQ helicases; however, a genetic disease has yet not been linked to mutations in the *RECQ1* gene, and the biological functions of human RECQ1 in cellular DNA metabolism are not known.

**Methodology/Principal Findings:**

In this study, we report that RECQ1 becomes phosphorylated upon DNA damage and forms irradiation-induced nuclear foci that associate with chromatin in human cells. Depletion of RECQ1 renders human cells sensitive to DNA damage induced by ionizing radiation or the topoisomerase inhibitor camptothecin, and results in spontaneous γ-H2AX foci and elevated sister chromatid exchanges, indicating aberrant repair of DNA breaks. Consistent with a role in homologous recombinational repair, endogenous RECQ1 is associated with the strand exchange protein Rad51 and the two proteins directly interact with high affinity.

**Conclusion/Significance:**

Collectively, these results provide the first evidence for a role of human RECQ1 in the response to DNA damage and chromosomal stability maintenance and point to the vital importance of RECQ1 in genome homeostasis.

## Introduction

It is believed that RecQ helicases have multiple roles in three facets of DNA metabolism (repair, replication and recombination), S-phase checkpoint, and telomere maintenance; consequently, they are considered caretakers of the genome [1], [2]. Three of the five human RecQ genes, designated *BLM, WRN* and *RECQ4,* have been genetically linked to the autosomal recessive diseases Bloom Syndrome (BS), Werner Syndrome (WS) and Rothmund-Thomson Syndrome (RTS), respectively. All three of these rare human diseases are characterized by a predisposition to cancer and chromosome instability, but the clinical features and cellular phenotypes are different from each other, suggesting unique roles of BLM, WRN, and RECQ4 helicases as tumor suppressors.

The biological significance of the remaining two human RecQ helicases, RECQ1 and RECQ5, is not yet known. The *RECQ1 (RECQL)* gene, originally cloned independently by two groups [3], [4], is located on chromosome 12p11-12 and encodes a 649 amino acid protein with a molecular mass of 73 kDa. RECQ1 was found to be the most abundant of the five human RecQ helicases in resting B cells, and its expression is upregulated in response to EBV transformation or treatment with the tumor promoting agent phorbol myristic acetate [5]. Despite the fact that RECQ1 was the first human RecQ helicase protein to be identified, little is known about its genetic functions in mammalian cells. Studies utilizing chicken DT40 cells have shown that RECQ1 and RECQ5 have roles in cell viability under a BLM-impaired condition, indicating a backup function for these helicases [6]. However, BLM, RECQ5, and RECQL have nonredundant roles in suppressing crossovers in mouse embryonic stem cells and fibroblasts [7], [8], suggesting a cell type or species-specific difference between the chicken and mouse systems.

In order to better understand and appreciate the molecular-genetic functions of RECQ1, its biochemical properties and protein interactions have been studied. RECQ1 is a nonprocessive helicase that unwinds dsDNA with a 3′ to 5′ polarity [9]. RECQ1 preferentially unwinds forked duplex substrates and two homologous recombination (HR) intermediates, the four-stranded Holliday Junction and three-stranded D-loop [10]. In addition to its helicase activity, RECQ1 efficiently catalyzes strand annealing of complementary ssDNA molecules in a reaction that is modulated by nucleotide binding to RECQ1 [10]. It was recently shown that RECQ1 assumes different oligomeric forms to perform its helicase and strand annealing activities [11]. The human single-stranded DNA binding protein Replication Protein A (RPA) modulates RECQ1 catalytic activities. By binding to ssDNA, RPA inhibits RECQ1 strand annealing [10]. In contrast, RPA stimulates RECQ1 unwinding activity in a specific manner which is likely mediated by a physical interaction with the RPA70 subunit [12]. RECQ1 binds to human mismatch repair factors (MSH2-MSH6, MLH1-PMS2, EXO-1) [13]. A functional importance for the physical protein interactions was evidenced by the abilities of RECQ1 to stimulate the endo- and exo-nucleolytic incision activities of EXO-1 and MSH2-MSH6 to enhance RECQ1 helicase activity [13]. RECQ1 was also reported to be associated with the Type IA Topoisomerase IIIα (Topo IIIα) in human cells [14]. Although the catalytic activities and protein interactions of RECQ1 suggest its involvement in the regulation of genetic recombination at some level, the precise roles of RECQ1 in cellular DNA metabolism are not understood.

In this study, we have investigated the potential importance of human RECQ1 in the DNA damage response and maintenance of chromosomal stability. In response to ionizing radiation (IR)-induced DNA damage, RECQ1 helicase undergoes sub-nuclear redistribution, protein phosphorylation, and preferential association with chromatin. RECQ1 depletion by RNA interference resulted in reduced cell proliferation, compromised cellular resistance to IR or camptothecin (CPT), and elevated sister chromatid exchange (SCE) in cells either untreated or exposed to CPT, suggesting that RECQ1 is important for the repair of endogenous or exogenously induced DNA damage.

## Materials and Methods

### Recombinant proteins

Recombinant human RECQ1 helicase was overexpressed in insect cells using a baculovirus encoding recombinant RECQ1 kindly provided by Dr. Alessandro Vindigni (International Center for Genetic Engineering and Biotechnology, Trieste, Italy) and purified as previously described [10]. Rad51 was generously provided by Dr. Ian Hickson (Cancer Research UK Laboratories).

### Cell lines and culture conditions

Human HeLa and U2OS cells were grown in Dulbecco's modified Eagle's medium (DMEM) (GIBCO-BRL, Carlsbad, CA) supplemented with 10% fetal bovine serum (Hyclone Laboratories, Logan, UT), 100 U/ml penicillin and 100 µg/ml streptomycin (Life Technologies, Carlsbad, CA). Cells were grown in a humidified 5% CO_2_ incubator at 37°C.

### RECQ1 silencing in HeLa cells

For siRNA mediated silencing of RECQ1, the siRNA duplexes were 19 base pairs with a 2-base overhang (Dharmacon Research, Chicago, IL). The sequences (sense strand) of RECQ1 siRNAL1 and siRNAL2 oligonucleotides are GCAAGGAGAUUUACUCGAAUU and GAAGAUUAUUGCACACUUUUU, respectively. The control siRNA has the sequence UAGCGACUAAACACAUCAAUU. Cells were transfected with siRNA duplexes by using Oligofectamine (Invitrogen, Carlsbad, CA) following the manufacturer's instructions. For constitutive silencing of RECQ1 expression in HeLa cells, two expression vectors containing short hairpin RNA (shRNA) sequences directed against RECQ1 (Origene, Rockville, MD) and a puromycin-resistance gene were transfected into HeLa cells using Fugene6 (Roche, Mannheim, Germany) according to the manufacturer's protocol. A control shRNA vector which has no homology to any human sequence was used as a control. 3-days post-transfection, cells were maintained in DMEM growth medium containing 10% serum and 0.5 µg/ml puromycin. Two sets of clones, each expressing one of the two shRNAs directed against RECQ1, were then used for further experiments.

### Cell proliferation analyses

Cells (24 h after transfection with either the control or two different RECQ1 siRNAs) were seeded in quadruplicate at a density of 300 cells/well in 96-well plates and allowed to adhere for 16 h. Cell proliferation using CyQuant (Molecular Probes, Eugene, OR) was assayed at 24, 48, 72, 96 and 120 h after transfection. As an indication of cell growth, total DNA was quantified at selected time points using a Fluorstar plate reader (B&L Systems, Maarssen, Netherlands) from a standard curve performed for each assay according to the manufacturer's instructions [15], [16]. The effects of RECQ1-siRNA on cell viability or growth were further determined by 3-(4, 5-Dimethyl-2-thiazolyl)-2,5-diphenyl-2H-tetrazolium bromide (MTT) assay. 48 h after siRNA transfection, cells were plated onto 96-well plates at a density of 5×10^3^ per well and incubated for 16 h. The medium was replaced with 100 µl of MTT solution and incubated for 1 h. After incubation, the MTT solution was removed and cells were suspended in 100% ethanol. Absorbance was measured at 590 nm using a microplate reader (Bio-Rad Laboratories, Hercules, CA). For the quantitative determination of DNA synthesis rate, mitogenic activity was determined by measuring BrdU incorporation using BrdU Cell Proliferation ELISA (Roche Diagnostics, Indianapolis, IN). Briefly, 48 h after siRNA transfection, 5×10^3^ cells were seeded in quadruplicates in 96-well plates and allowed to adhere for 16 h. Cells were then labeled for 3 h with bromodeoxyuridine, and DNA synthesis was measured with the ELISA BrdUrd assay according to the manufacturer's instructions. The absorbance values correlate directly to the amount of DNA synthesis and the number of proliferating cells in culture. Absorbance was measured at 450 nm with a microplate reader (Bio-Rad Laboratories, Hercules, CA). All cell proliferation assays were performed at least four times.

### Clonogenic and survival assays of shRNA-treated cells

To analyze the relative growth rate of the shRNA treated cells, 5×10^3^ cells were seeded in a 24-well plate in triplicate and the total cell number was counted at indicated time points using a Coulter counter (Beckman, Fullerton, CA). For the clonogenic assays, 500 cells were reseeded in 6-well plates. The number and size of methylene blue-stained colonies were recorded after 5–9 days of growth in complete medium.

### Cell cycle analysis

HeLa cells were harvested by trypsinization 72 h after transfection and fixed with 70% ethanol. After RNase treatment, cells were stained with propidium iodide, and DNA histograms were acquired using a FACScalibur. Cell cycle distributions were modeled using Multicycle (Phoenix Flow Systems, San Diego, CA).

### Analysis of cellular apoptosis

To assay the endogenous spontaneous cellular apoptosis, cells were treated with either the control or RECQ1 siRNA duplexes for 48 h. Subsequently, the cytoplasmic histone-associated DNA fragments, which are indicative of ongoing apoptosis, were quantitatively measured by using the Cell Death Detection ELISAPLUS photometric enzyme-immunoassay method (Roche Applied Science, Indianapolis, IN) according to the manufacturer's instructions. To assay cell sensitization and the extent of apoptosis induction after DNA damage or oxidative stress, an equal number of siRNA-treated cells were subcultured in complete medium containing 400 µM H_2_O_2_ for 3 h followed by a recovery period in complete medium for 21 h. Apoptosis induction was then quantified by using the ELISAPLUS death assay. Western detection of PARP-1 cleavage was performed using monoclonal mouse anti-PARP-1 antibody (2 µg/ml; BD Biosciences PharMingen, San Diego, CA) recognizing both cleaved and uncleaved PARP-1.

### DNA damage survival assays

Survival assays utilizing siRNA transfected HeLa cells were performed 48 h after the transfection. Cells (transfected with either the control or two different RECQ1 siRNAs) were seeded in quadruplicate at a density of 300 cells/well in 96-well plates, allowed to adhere for 14–16 h, and subsequently exposed to increasing doses of IR using Gammacell 40 (Nordion International, Inc.), a ^137^Cs source emitting at a fixed dose rate of 0.82 Gy/min. To determine CPT sensitivity, cells were exposed continuously to increasing concentrations of CPT (Sigma, St. Louis, MO). Following treatment, cells were allowed to grow at 37°C for 5 days in 5% CO_2_. Plates were frozen at −80°C. Total DNA was quantified using CyQuant (Molecular Probes, Eugene, OR) and compared with untreated controls as an indication of cell growth as described [15], [16]. Quantification of DNA using CyQuant was performed using a Fluorstar plate reader (B&L Systems) according to the manufacturer's instructions.

### Radioresistant DNA synthesis and G_2_/M checkpoint assays

Cells transfected with control or RECQ1 siRNA were used for radioresistant DNA synthesis assays 48 h after transfection. Cells were labeled for 24 h with 10 nCi/ml [^14^C]thymidine and then incubated for another 24 h in non-radioactive medium. Thirty min after irradiation (10 Gy), 2.5 µCi/ml [^3^H]thymidine was added to the medium for 15 min to allow cell labeling. The cells were collected and transferred to Whatman filters and fixed sequentially with 70% methanol followed by 95% methanol. Radioactivity was measured in a liquid scintillation counter. The measure of DNA synthesis was derived from resulting ratios of [^3^H]/[^14^C] and expressed as a percentage of control values. For G_2_/M checkpoint assays, HeLa cells were transfected with siRNAs and irradiated 48 h after transfection. Cells were harvested 1 h after irradiation, ethanol-fixed, stained with propidium iodide and anti-phosphohistone H3 antibodies followed by Alexa Fluor 488-conjugated secondary antibody (Molecular Probes, Carlsbad, CA), and analyzed using a FACScalibur.

### Sister chromatid exchange assays

SCEs were detected as described [17]. At 36 h after HeLa cells were transfected with siRNA oligos, cultures were grown in the presence of 100 µM of 5-bromodeoxyuridine (BrdU; Sigma) through two cell cycles to achieve preferential labeling of sister chromatids. Colcemid (Sigma) was added at a final concentration of 0.05 µg/ml to accumulate mitotic cells 2 h prior to harvesting cells. Harvested cells were then incubated in hypotonic solution (0.06 M KCl) for 15 min at room temperature, and then fixed with 3∶1 (vol/vol) methanol–glacial acetic acid. Fixed cell suspension was dropped onto a glass slide and air-dried. Slides were aged for 3 days and stained with 5 µg/ml Hoechst 33258 (Sigma) for 10 min. Sister chromatid differentiation was performed by the fluorescence-plus-Giemsa technique [17]. For Mitomycin C (MMC) treatment, MMC was added to the culture at a final concentration of 60 nM for 16 h prior to harvesting. For CPT treatment, cells were exposed to 1 µM CPT for 1 h, washed twice, and recovered in drug-free media containing BrdU for two cell cycles before harvesting. Digitally captured images of at least 25 differentially stained metaphase chromosome spreads per treatment were scored in a blinded fashion.

### *In situ* fractionation and immunofluorescence detection of endogenous RECQ1

HeLa cells grown on glass coverslips in 35-mm dishes to about 70% confluence were untreated or treated with IR (10 Gy). Six h after IR treatment, the cells were processed for immunofluorescent staining. For detection of chromatin bound protein, the *in situ* cell fractionation procedure was performed before fixation [18]. Briefly, coverslips were washed in phosphate-buffered saline (PBS), incubated in cytoskeleton buffer (10 mM PIPES (pH 6.8), 100 mM NaCl, 300 mM sucrose, 3 mM MgCl_2_, 1 mM EGTA, 0.5% Triton X-100) for 5 min on ice, followed by incubation in cytoskeleton stripping buffer (10 mM Tris-HCl (pH 7.4), 10 mM NaCl, 3 mM MgCl_2_, 1% Nonidet P-40, 0.5% sodium deoxycholate) for 5 min on ice. After several washes with ice-cold PBS, the cells were fixed with 3.7% paraformaldehyde for 10 min and permeabilized in 0.5% Triton X-100 solution for 10 min at room temperature. Intact cells (not extracted with Nonidet P-40) were fixed as described above. Cells were blocked with 10% FCS in PBS and incubated with rabbit polyclonal anti-RECQ1 antibody (1∶1000, Santa Cruz Biotech, Santa Cruz, CA) overnight at 4°C in a humid chamber. Following five washes with 0.1% Triton X-100 in 1X PBS, cells were incubated with rhodamine conjugated goat anti-rabbit IgG (1∶500, Vector Laboratories, Burlingame, CA) for 1 h at 37°C. All antibodies were diluted in 5% FCS–PBS. After washing five times, coverslips were mounted with Vectashield mounting medium containing DAPI (Vector Laboratories) and analyzed by fluorescence microscopy. Images were captured on an AxioVision 3.1 microscope (Zeiss) with deconvolution using a black and white CCD camera. Representative photographs from three independent experiments are shown in the figures.

### Biochemical fractionation

U2OS cells that were either untreated or exposed to IR (10 Gy) and allowed to recover for 6 h were trypsinized and collected by centrifugation. Cells were washed once with cold PBS and portioned equally in four eppendorf tubes, and their sub-cellular fractionation was performed as described [19]. Briefly, following centrifugation at 4°C for 3 min at 300 *g*, one pellet which represented the whole cell pellet (P1) was frozen in liquid nitrogen and the remaining pellets were resuspended in cold buffer A (10 mM PIPES (pH 7.0), 100 mM NaCl, 3 mM MgCl_2_, 1 mM EGTA, 300 mM sucrose, 0.5 mM Na_3_VO_4_, 50 mM NaF, 10 µg/ml aprotinin, 10 µg/ml leupeptin, 10 µg/ml pepstatin A, and 1 mM phenylmethylsulfonylfluoride (PMSF)) containing 0.5% Triton X-100 and incubated at room temperature for 2 min to permeabilize cells. Following centrifugation at 4°C for 3 min at 300 *g*, the supernatant which represented cytosol and nucleosol fractions (S2) was collected and frozen. The pellet was washed with cold buffer A. One pellet which represented detergent-insoluble nuclei (P2) was frozen. The detergent insoluble nuclei from the other pellet were then digested with RNase-free DNase I (200 U/ml, New England Bio-Labs, Ipswich, MA) in buffer A for 30 min at room temperature followed by centrifugation at 4°C for 3 min at 300 *g*. The supernatant (S3) was frozen, and the pellet that had been washed with cold buffer A was either frozen (P3) or extracted with cold buffer A containing 0.25 M ammonium sulfate for 5 min at room temperature to solubilize the remaining chromatin. The residual pellet which represented the nuclear matrix fraction (P4) was collected by centrifugation at 4°C for 3 min at 1877 *g*, and the supernatant which contained chromatin (S4), as identified by the presence of histones, was frozen. The pellet was frozen following a single wash with cold buffer A and centrifugation at 800 *g* for 3 min. Proteins from equivalent cell volumes of each fraction step were resolved by 10% SDS-PAGE and probed for RECQ1 (1∶750, Santa Cruz Biotech, Santa Cruz, CA), histone H4 (chromatin marker, 1∶5000, Upstate Biotech, Upstate, NY), and lamin B (nuclear matrix marker, 1∶1000, Abcam, Cambridge, MA) by immunoblotting.

### RECQ1 immunoprecipitation and phosphatase treatment

Whole cell extracts were prepared in lysis buffer (50 mM Tris-HCl (pH 7.4), 150 mM NaCl, and 1% (v/v) Triton X-100) supplemented with protease inhibitors (1 µg/ml leupeptin and pepstatin, 2 µg/ml aprotinin, and 1 mM PMSF) and phosphatase inhibitors (1 mM Na_3_VO_4_ and 10 mM NaF). Using a polyclonal antibody against human RECQ1 (Santa Cruz Biotech), immunoprecipitation (IP) was performed essentially as previously described [13]. Each RECQ1 IP (normalized to contain 0.25 mg of protein as the input) was washed twice in lambda phosphatase buffer (New England BioLabs) and resuspended in 50 µl of lambda phosphatase buffer either in the presence or absence of lambda phosphatase (500 U, New England BioLabs), followed by incubation at 30°C for 1 h. Proteins were resolved by 12% SDS-PAGE and immunoblotted for RECQ1 using rabbit polyclonal RECQ1 antibody (1∶750, Santa Cruz Biotech). For the detection of phosphorylated RECQ1 in soluble and chromatin-bound fractions, total soluble and chromatin-bound fractions were prepared from aliquots of 1×10^7^ cells and analyzed by immunoprecipitation and Western as described above.

### ELISA

Wells were coated with 50 µl (1 ng/µl) of either BSA or RECQ1 protein diluted in carbonate buffer (0.016 M Na_2_CO_3_, 0.034 M NaHCO_3_ (pH 9.6)), and incubated at 4°C overnight. The samples were aspirated, and the wells were blocked for 2 h at 30°C with blocking buffer (PBS, 0.5% Tween-20, 3% BSA). The wells were washed with PBS containing 0.5% Tween-20. Serial dilutions of Rad51 in blocking buffer were then added to the appropriate wells of the ELISA plate and incubated for 1 h at 30°C. DNaseI (100 U/ml) or ethidium bromide (EtBr) (50 µg/ml) was included in the incubation with Rad51 in the binding step in the corresponding wells to test for DNA mediated protein interaction. The samples were aspirated, and the wells were washed five times with washing buffer before addition of rabbit anti-Rad51 antibody (Calbiochem, San Diego, CA) diluted 1∶500 in blocking buffer and incubated at 30°C for 1 h. Following three washes with washing buffer, HRP-conjugated anti-rabbit secondary antibody diluted 1∶10,000 in blocking buffer was added to the wells, and the samples were incubated for 30 min at 30°C. After washing five times, any Rad51 bound to the RECQ1 was detected using OPD substrate (Sigma). The reaction was terminated after 3 min with 3 N H_2_SO_4_ and absorbance readings were taken at 490 nm.

### Co-immunoprecipitation experiments

Nuclear extracts were prepared from exponentially growing HeLa cells as described previously [20]. Nuclear extract (1 mg of total protein) was incubated with either rabbit polyclonal anti-RECQ1 (2 µg, Santa Cruz Biotech) or mouse monoclonal anti-Rad51 antibodies (2 µg, Oncogene) in buffer D (50 mM HEPES (pH 7.5), 100 mM KCl, 10% glycerol) for 4 h at 4°C. The mixture was subsequently tumbled with 20 µl of protein G-agarose (Roche Applied Science) at 4°C overnight. The beads were then washed three times with buffer D supplemented with 0.1% Tween 20. Proteins were eluted by boiling in SDS sample buffer, and half of the eluate was resolved on 10% polyacrylamide Tris-glycine SDS gels, and transferred to polyvinylidene difluoride membranes (Amersham Biosciences, Piscataway, NJ). The membranes were blocked with 5% nonfat dry milk in PBS containing 0.1% Tween 20, and probed for RECQ1 and Rad51 using anti-rabbit RECQ1 (1∶750, Santa Cruz Biotech) and anti-rabbit Rad51 (1∶1000, Calbiochem) antibodies respectively, followed by detection with a goat-anti-rabbit IgG secondary antibody conjugated to horseradish peroxidase (HRP) (Santa Cruz Biotechnology). RECQ1 and Rad51 on immunoblots were detected using ECL Plus (Amersham Biosciences).

## Results

Of the five human RecQ helicases, RECQ1 is one of the least well characterized in terms of its biological roles. Although RECQ1 is not implicated as of yet in a human disease, studies of cells from RECQL knockout mice suggested a role of RECQ1 in the maintenance of chromosomal stability and the repair of double strand breaks [8]. Other human RecQ helicases, particularly WRN and BLM, have been proposed to participate in pathways which help cells to deal with replicational stress or repair DNA damage [2]. Thus, we set out to characterize the importance of human RECQ1 in the DNA damage response in order to obtain an understanding of its biological relevance in cellular DNA metabolism.

### DNA damage induced relocalization of nucleolar RECQ1 helicase

**Figure 1:**
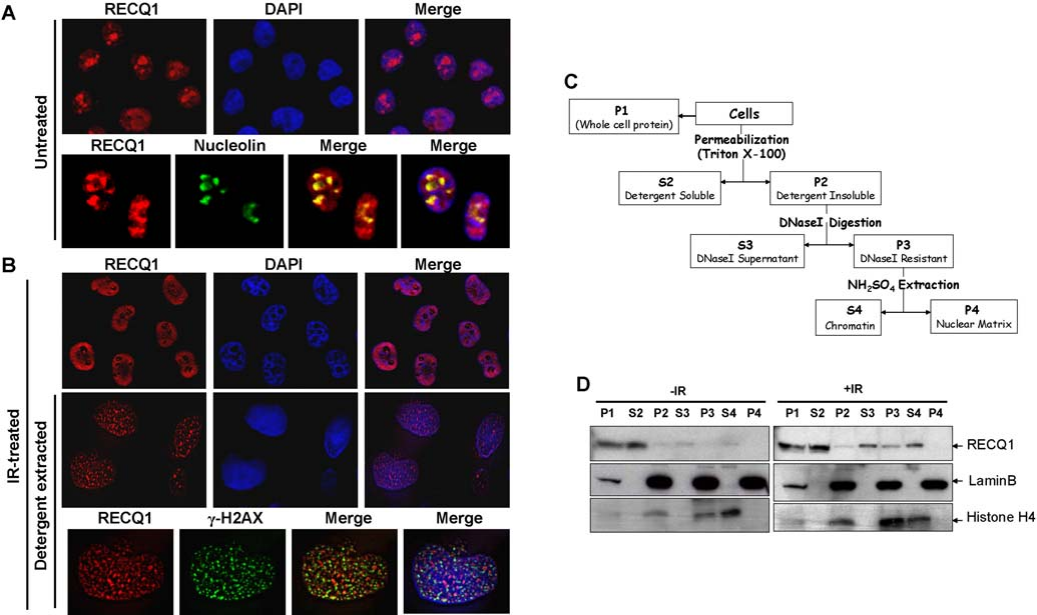
Nucleolar RECQ1 undergoes re-localization to chromatin in response to IR. Indirect immunofluorescence was performed on HeLa cells that were either untreated or allowed to recover for 6 h from 10 Gy IR exposure as described in “Materials and Methods”. *Panel A*, Nucleolar localization of RECQ1 in untreated cells. Cells were co-immunostained with anti-RECQ1 and anti-nucleolin. The merged images show cells stained with RECQ1 (red) and nucleolin (green), with or without DAPI (blue). *Panel B,* Relocalization of RECQ1 to chromatin-bound foci following IR damage. After IR exposure, cells were stained for total RECQ1 (top panel) or *in situ* detergent extraction-resistant RECQ1 (middle and bottom panel). Merged images show RECQ1 (red) and DAPI (blue). Co-immunostaining of chromatin-bound RECQ1 and γ-H2AX (*Panel B, bottom*). Merged images show RECQ1 (red) and γ-H2AX (green), with or without DAPI (blue). *Panel C,* Schematic presentation of the protocol for sequential nuclear fractionation of lysates from U2OS cells either untreated or following 6 h recovery from 10 Gy IR exposure. Cytoplasmic and nucleoplasmic proteins were extracted by permeabilization with detergent, and the resulting nuclei were nuclease-digested and extracted with NH_2_SO_4_. Proteins of the supernatant (S) and pellet (P) fractions were resolved on 10% SDS-PAGE, and subsequently analyzed by Western blot for RECQ1, histone H4 (chromatin marker), and lamin B (nuclear matrix marker). *Panel D,* Following IR treatment, RECQ1 is enriched in the chromatin fraction (S4).

First, we examined by immunofluorescence the sub-cellular distribution of endogenous RECQ1 in human cells either untreated or exposed to IR which directly introduces DNA strand breaks. In untreated HeLa cells, RECQ1 displayed nuclear staining with a predominant localization in nucleoli (Fig. 1A). Nucleolar localization of RECQ1 in untreated cells was confirmed by co-staining with nucleolin (Fig. 1A), consistent with its identification in the nucleolar proteome [21], [22]. Cellular exposure to IR (10 Gy) resulted in the loss of RECQ1 nucleolar staining and the appearance of RECQ1 staining in the nucleoplasm (Fig. 1B). Approximately 70–80% of the cells showed exclusion of RECQ1 from the nucleolus to the nuclear compartment within 6 h after IR exposure.

Since the more well characterized BLM helicase has been implicated in the cellular response to replicative stress [23]–[26], we next assessed if relocalization of RECQ1 may also be elicited by cellular exposure to the replication inhibitor hydroxyurea. The sub-nuclear distribution of RECQ1 is altered upon hydroxyurea treatment (Fig. S1A), suggesting that RECQ1 relocalization may be a general response to DNA damage or replicational stress.

### RECQ1 associates with chromatin upon IR exposure

We next sought to determine if endogenous RECQ1 would be associated with damaged DNA. *In situ* fractionation based on successive detergent extractions displayed punctuate pattern of nuclear retention of the DNA bound RECQ1 upon IR exposure (Fig. 1B). In order to determine the retention of RECQ1 at the sites of double strand breaks, we looked for the co-localization of RECQ1 with γ-H2AX and found only a small fraction of extraction-resistant RECQ1 aggregates co-localized with γ-H2AX foci (Fig. 1B).

Nuclear proteins from U2OS cells either untreated or harvested 6 h following IR (10 Gy) exposure were fractionated into soluble nuclear, chromatin, or nuclear matrix components (Fig. 1C), and fractions were immunoblotted for RECQ1 (Fig. 1D). RECQ1 protein fractionated with soluble nuclear proteins (Fig. 1D, S2 fraction) from the untreated cells, with only a very minor fraction associated with chromatin (Fig. 1D, S4 fraction). Following IR treatment, RECQ1 was also found in the insoluble fractions (Fig. 1D), and a significantly greater amount of RECQ1 fractionated with chromatin that also contained histones (Fig. 1D, S4 fraction). RECQ1 was not visibly detected in the nuclear matrix fraction (P4), a fraction that contains lamin B (Fig. 1D). These results indicate that γ-irradiation of human cells targets RECQ1 to associate with chromatin.

### Endogenous RECQ1 is phosphorylated in response to DNA damage

**Figure 2:**
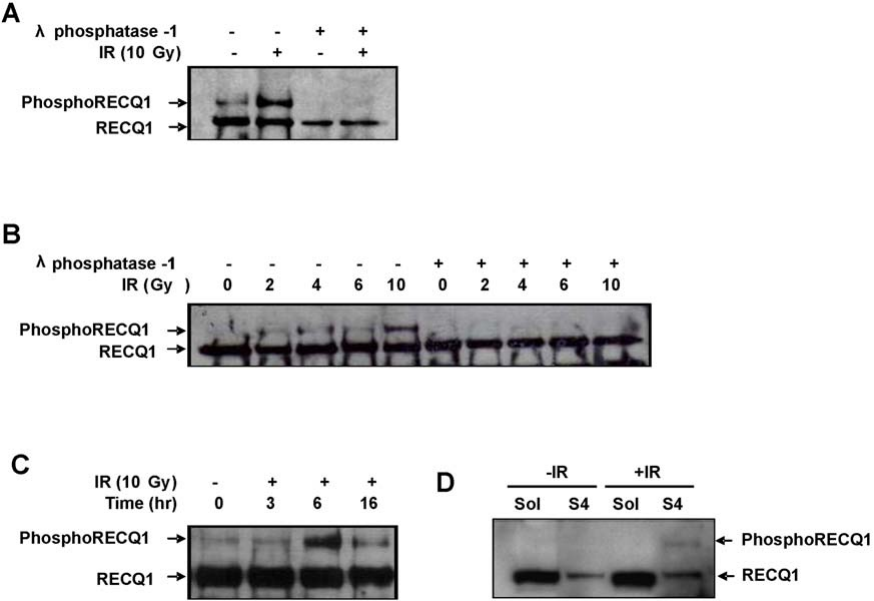
Phosphorylation of endogenous RECQ1 in response to IR. *Panel A,* RECQ1 was immunoprecipitated from whole cell extracts of U2OS cells that were either untreated or allowed to recover for 6 h from 10 Gy IR exposure. The RECQ1 immunoprecipitate from each cell extract was divided in half and was either incubated or not with λ-phosphatase (500 U) for 1 h at 30°C before elution with SDS sample buffer. The immunoprecipitated proteins were resolved on 12% SDS-PAGE, transferred to PVDF membranes, and probed for RECQ1. *Panel B,* IR-induced RECQ1 phosphorylation is dependent on radiation dose. RECQ1 was immunoprecipitated from either untreated cells or 6 h after treatment of cells with the indicated dose of γ-radiation. Immunoprecipitated proteins were divided in two aliquots which were either treated with λ-phosphatase or left untreated. RECQ1 was detected as described above. *Panel C,* Time course of RECQ1 phosphorylation in response to IR. U2OS cells were subjected to γ-radiation (10 Gy), and RECQ1 was immunoprecipiated from whole cell extracts prepared at the indicated time points following IR exposure. *Panel D,* Phosphorylated RECQ1 is preferentially associated with chromatin in IR-treated cells. RECQ1 was immunoprecipitated from the detergent-soluble (S2) and insoluble (S4) fractions of untreated or IR treated cells, resolved on 12% SDS-PAGE, and detected by Western blot analysis.

A critical element of the DNA damage response is the activation of signaling pathways by protein phosphorylation to initiate repair by HR [27], [28]. To assess if RECQ1 might be post-translationally modified after DNA damage, RECQ1 was immunoprecipitated from protein extracts prepared from cells that were either untreated or γ -irradiated (10 Gy) and harvested 6 h following exposure. Western blot using anti-RECQ1 antibody demonstrated two differentially migrating bands of RECQ1 in cell extracts with the intensity of the slower migrating band much stronger in the immunoprecipitates from the IR treated cells (Fig. 2A, lanes 1, 2). Treatment of immunoprecipitated RECQ1 with lambda protein phosphatase resulted in disappearance of the slower migrating band (Fig. 2A, lanes 3, 4). Lambda phosphatase treatment of the RECQ1 immunoprecipitates in the presence of phosphatase inhibitors did not affect the mobility of the slower migrating band (data not shown). These results indicate that the RECQ1 mobility shift observed in response to IR was due to protein phosphorylation. This IR-induced phosphorylation of RECQ1 was found to be both dependent on irradiation dose and time (Fig. 2B, 2C). The slower migrating band corresponding to phosphorylated RECQ1 was detected in immunoprecipitates from cells irradiated with a low IR dose of 2 Gy (Fig. 2B), and maximal phosphorylation of endogenous RECQ1 was obtained in 6 h after 10 Gy irradiation (Fig. 2C). RECQ1 immunoprecipitates from the soluble (S2) and insoluble (S4) fractions from cells either untreated or γ-irradiated (10 Gy, 6 h following exposure) demonstrated a slow migrating band in the chromatin-associated fraction (Fig. 2D), indicating that phosphorylated RECQ1 is preferentially associated with chromatin upon IR exposure. RECQ1 was also phosphorylated in response to ultraviolet light or the replication inhibitor hydroxyurea (Fig. S1B).

### RECQ1 depletion results in a decrease of cellular growth and proliferation

**Figure 3:**
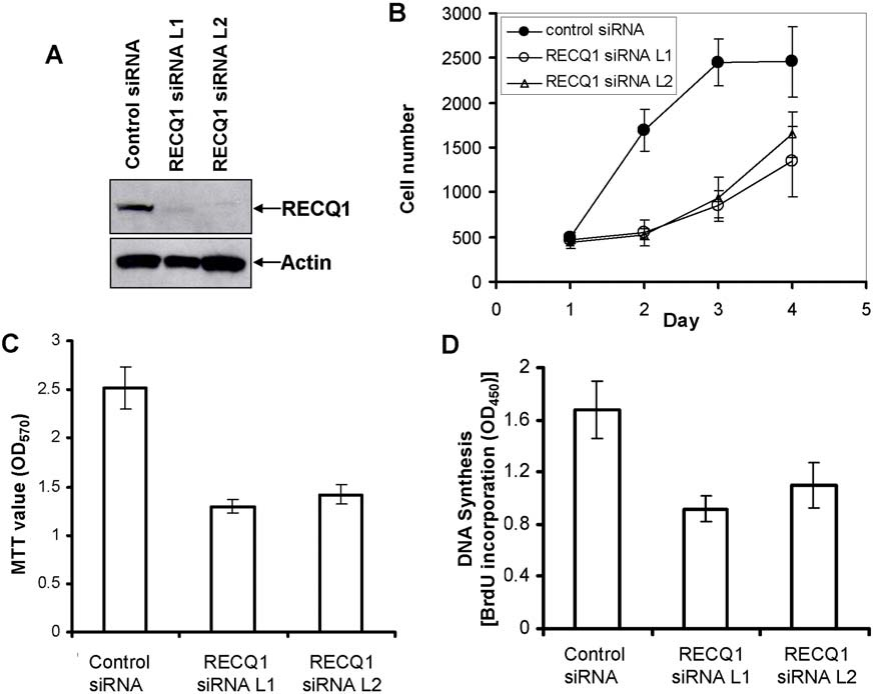
RECQ1-depleted cells show reduced cellular proliferation. *Panel A*, Small interfering RNA inhibition of RECQ1. Whole cell extracts from HeLa cells that had been transfected with control or RECQ1 siRNA (oligonucleotide L1 or L2) were subjected to Western blotting with RECQ1 or Actin antibodies (as a loading control). *Panel B*, Proliferation of control or RECQ1 siRNA treated cells as determined by CyQuant assay at indicated time points after transfection. *Panel C*, MTT assay of *in vitro* proliferation of control or RECQ1-siRNA transfected cells. *Panel D*, Colorimetric BrdU cell proliferation ELISA of control or RECQ1 siRNA treated HeLa cells. Results are taken from three independent experiments and proliferation is represented as the mean±standard deviation (SD).

The proposed roles of RecQ helicases in replication fork processing suggested that RECQ1 status may influence cellular growth and proliferation. Since an effect of RECQ1 deficiency on growth properties had not been previously examined, we evaluated several biological parameters pertaining to growth and proliferation in human cells depleted of RECQ1 by RNA intereference. siRNA depletion of RECQ1 during a 48 h time period led to a decrease of >90% of RECQ1 compared to control as observed by Western blotting (Fig. 3A). Beginning 48 h after siRNA treatment, total DNA content was evaluated at 24 h time intervals as a measure of cell proliferation. The results, shown in Fig. 3B, demonstrate that RECQ1 depletion by either the siRNAL1 or siRNAL2 oligonucleotides resulted in a statistically similar reduction in total DNA content compared to cells treated with the control siRNA. At day 2, total DNA content was increased over 3-fold in control siRNA transfected cells whereas there was statistically no increase in DNA content in the siRNA RECQ1-depleted cells. At day 3, the control cultures had increased their DNA content 5-fold. In contrast, the DNA content of RECQ1-depleted cells increased only 1.5-fold. Measurable differences in metabolic activity between the control and RECQ1-depleted cells were detected using the MTT assay (Fig. 3C), consistent with the observed decrease in cellular DNA content. To test the mitogenic efficiency of RECQ1-depleted cells, we evaluated BrdU incorporation as a measure of DNA synthesis. The results from these experiments, shown in Fig. 3D, demonstrate that RECQ1-depleted cells are impaired for their ability to synthesize DNA as compared to control siRNA treated cells. Taken together, these results suggest that RECQ1 has a regulatory role in cell proliferation.

**Figure 4:**
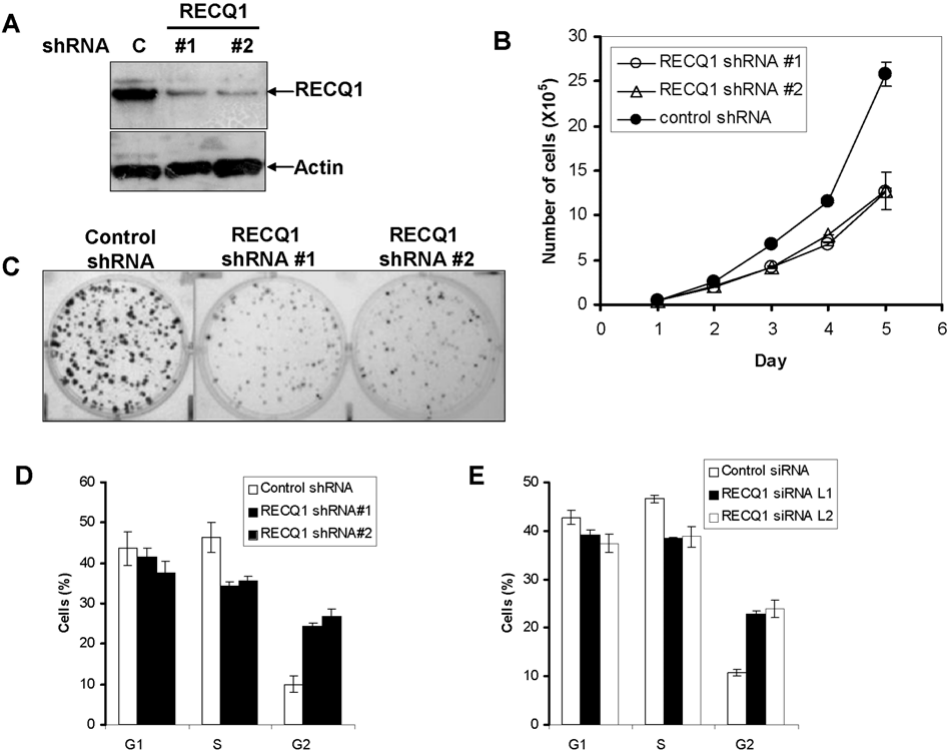
Reduced cell growth and aberrant cell cycle progression of RECQ1-depleted cells. *Panel A*, Short hairpin RNA (shRNA)-mediated RECQ1 depletion in HeLa cells. Western blot showing RECQ1 expression in puromycin resistant HeLa cells transfected with either control or RECQ1 shRNA (#1 or #2). Actin is used as loading control. Proliferation of control or cells transfected with RECQ1-specific shRNA plasmids was determined by Coulter counting the total number of cells at indicated time points (*Panel B*) and by colony forming assay (*Panel C*). *Panels D* and *E*, RECQ1 depletion induces G2/M accumulation. Flow cytometry was used to determine cell cycle distribution of control or RECQ1 shRNA transfected cells (*Panel D*) or RECQ1 siRNA transfected cells (*Panel E*).

To evaluate the importance of RECQ1 for cell growth, HeLa cells were transfected with a plasmid encoding shRNA against RECQ1 or a control plasmid. This approach enabled us to assess the effect of RECQ1 depletion on cell number and colony forming ability. Efficient depletion of RECQ1 protein was observed in the whole cell extracts of puromycin resistant cultures that harbor RECQ1 shRNA plasmid as compared to the control (Fig. 4A). To determine the proliferative survival of RECQ1-depleted cells, an equivalent number of puromycin resistant cells were plated and counted sequentially for 5 days using a Coulter counter. Cells that had been transfected with plasmid encoding RECQ1 shRNA displayed reduced cell numbers compared to the control cells on days 3, 4 and 5 (Fig. 4B). Colony forming assay displayed a significant reduction in both the size and number of colonies when cells were inhibited for the expression of RECQ1 (Fig. 4C). RECQ1-depleted HeLa cells reproducibly displayed an increased (>2-fold) percentage of cells in the G2 phase compared to the control cells (Fig. 4D and 4E), suggesting perturbation of normal cell cycle progression.

### Spontaneous and DNA damage-induced apoptosis of RECQ1-depleted cells

**Figure 5:**
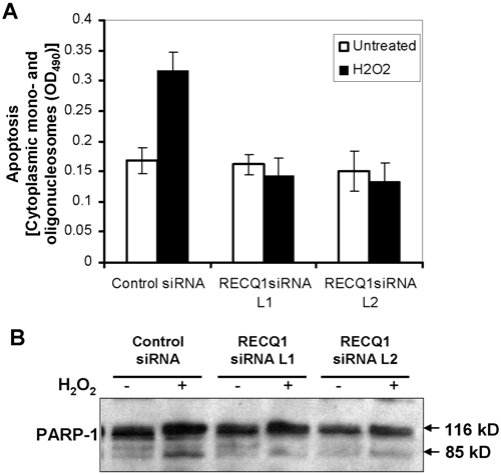
Effect of RECQ1 depletion on spontaneous or oxidative stress induced apoptosis. HeLa cells were siRNA treated for 48 h, and then incubated in complete medium for 24 h. Cells were either untreated or incubated with 400 µM H_2_O_2_ for 3 h in serum free medium, washed, and allowed to recover in complete medium for 21 h. *Panel A,* Enrichment of the cytoplasmic histone-associated-DNA fragments that are indicative of an ongoing apoptosis in cells with the indicated siRNA oligonucleotides. Samples were analyzed in duplicates, and data points represent the mean of three independent experiments; *bars* denote SD. *Panel B,* immunoblotting analysis of the PARP cleavage in control or RECQ1 siRNA treated HeLa cells either untreated or exposed to 400 µM H_2_O_2_.

Programmed cell death by the apoptotic pathway is an important component of the cellular response to stress or DNA damage. Mutations in genes encoding RecQ helicases and other tumor suppressors (p53, promyelocytic leukemia (PML) protein) are defective in stress-induced apoptosis [29]–[33]. The negative effect of RECQ1 depletion on cellular proliferation raised the possibility that RECQ1 status might affect apoptotic potential. As shown quantitatively in Fig. 5A, there was no difference observed in apoptosis as measured by cytoplasmic mono- and oligo-nucleosomes between untreated control or RECQ1 siRNA treated cells; however, RECQ1 depleted cells exposed to 400 µM H_2_O_2_, a concentration previously shown to induce apoptosis [34], were compromised in their apoptotic response compared to control siRNA treated cells which doubled their level of apoptosis after H_2_O_2_ exposure. Similarly, PARP-1 cleavage was also reduced in RECQ1-depleted cells after H_2_O_2_ exposure compared to the control cells (Fig. 5B). These results suggest that cellular depletion of RECQ1 does not induce apoptosis; however, RECQ1 plays a role in the apoptotic response to H_2_O_2_-induced stress which induces oxidized bases, single-stranded DNA breaks, and double-stranded DNA breaks.

### RECQ1 depletion leads to increased sensitivity to genotoxic stress

**Figure 6:**
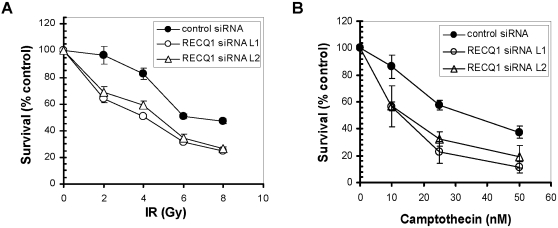
RECQ1 depletion sensitizes cells to DNA damage induced by ionizing radiation or camptothecin. siRNA knockdown of RECQ1 in HeLa cells leads to increased sensitivity to treatment with IR (*Panel A*) or CPT (*Panel B*). HeLa cells treated with either RECQ1 siRNA or control siRNA (#C) were plated in quadruplicate and treated with increasing doses of IR or concentrations of CPT. Total DNA content was measured as an indication of cell growth. Two independent siRNAs (#L1 and #L2) used to downregulate RECQ1 expression resulted in similar growth phenotype in HeLa cells. Three independent determinations of cell survival were performed and the mean±SD is presented.

The collective evidence suggesting a vital importance of RecQ helicases in the maintenance of genomic stability led us to investigate if human RECQ1 might play a unique role in the DNA damage response. The elevated IR sensitivity and sister chromatid exchange in RECQL knockout mouse cells [8] suggested a role of RECQ1 in HR repair. Therefore, we examined the IR sensitivity of human cells depleted of RECQ1. HeLa cells transfected with control or RECQ1 siRNA were exposed to increasing doses of IR, and cellular proliferation was assayed. As shown in Fig 6A, depletion of RECQ1 in HeLa cells rendered the cells sensitive to IR as compared to control siRNA treated HeLa cells.

Evidence that RecQ helicases function to preserve genomic stability at stalled or broken replication forks [2] prompted us to examine the sensitivity of RECQ1-depleted cells to CPT, an anti-tumor drug that inhibits the topoisomerase-induced DNA breakage-reunion reaction [35]. Topoisomerase I-DNA cleavable complexes are stabilized by a ternary interaction with CPT resulting in DSBs at stalled replication forks and cell death. Since CPT-induced double strand breaks at stalled replication forks are repaired by recombination, we postulated that RECQ1, like BLM helicase [36], may be important in the cellular resistance to CPT. As shown in Fig. 6B, RECQ1 depletion resulted in a significantly greater sensitivity to CPT at all drug concentrations tested (10, 25, 50 nM). Taken together these results indicate that depletion of RECQ1 compromises the ability of cells to respond and survive after IR or CPT-mediated genotoxic stress.

### Effect of RECQ1 depletion on G2/M and intra-S phase DNA damage checkpoint

**Figure 7:**
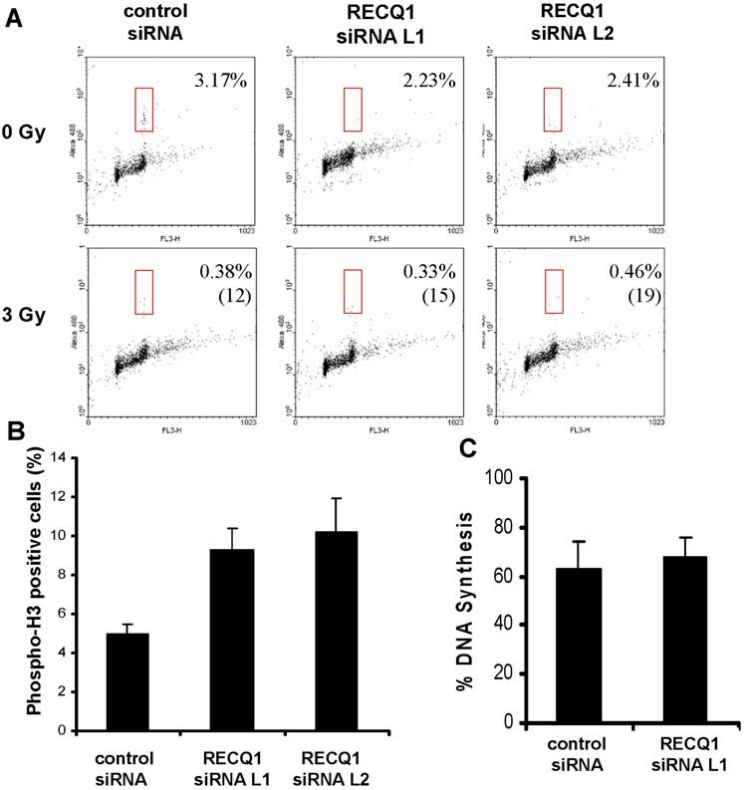
Effect of RECQ1 depletion on G2/M and intra-S-phase DNA damage checkpoints. *Panel A*, DNA damage-induced mitotic entry delay is minimally affected by the RECQ1 deficiency. HeLa cells were transfected with the indicated siRNAs and either mock-treated or exposed to 3 Gy of IR 1 h before harvesting. Mitotic cells were detected by PI and phosphohistone H3 staining and analyzed by flow cytometry. Percentages of mitotic cells and their levels normalized to control (in parentheses) are shown. *Panel B,* RECQ1 is involved in maintenance of IR-induced G2/M checkpoint. Cells with either control or RECQ1 siRNA were exposed to 10 Gy of IR. Nocodazole (1 µg/ml) was added to the medium at the time of IR treatment to capture cells entering mitosis. 16 h later, cells were collected for PI and phospho-histone H3 staining and analyzed by flow cytometry. *Panel C,* HeLa cells transfected with control or RECQ1 siRNAs were exposed to 10 Gy of IR and assayed for DNA synthesis 30 min later by [^3^H]thymidine incorporation. The amount of DNA synthesis after irradiation is expressed as a percentage of the level in untreated cells. Error bars indicate SD from three independent experiments.

To determine whether DNA damage signaling is compromised upon RECQ1 depletion, we examined the DNA damage-induced G2/M checkpoint in RECQ1 siRNA-treated cells. HeLa cells were transfected with the indicated siRNA and were either mock-treated or subjected to 3 Gy IR 48 h after transfection. Cells were harvested 1 h after treatment and analysed by flow cytometry for histone H3 phosphorylation as a marker for entry into mitosis. As expected, very few of the control siRNA-treated cells entered mitosis after 3 Gy of IR (Fig. 7A). Similarly, RECQ1 siRNA treated cells showed profound reduction of the phospho-H3 positive 4N population, suggesting that RECQ1 is not essential for establishment of the G2/M checkpoint.

To determine if RECQ1 is required for the maintenance of the G2/M checkpoint, cells were irradiated (10 Gy) and incubated in the presence of nocodazole for 16 h to capture cells entering mitosis. Cells depleted in RECQ1 yielded a significantly higher mitotic population, as reflected by the higher percentage of phospho-H3 positive 4N population, compared to the control siRNA transfected HeLa cells (Fig. 7B). Increased Phospho-H3 staining indicated that RECQ1 depletion compromises cellular ability to maintain IR-induced G2/M checkpoint. However, depletion of RECQ1 did not compromise the ability of cells to down-regulate DNA synthesis upon IR exposure (Fig. 7C), suggesting that RECQ1 does not play a major role in intra-S phase checkpoint.

### Depletion of RECQ1 leads to spontaneous formation of γ-H2AX foci

**Figure 8:**
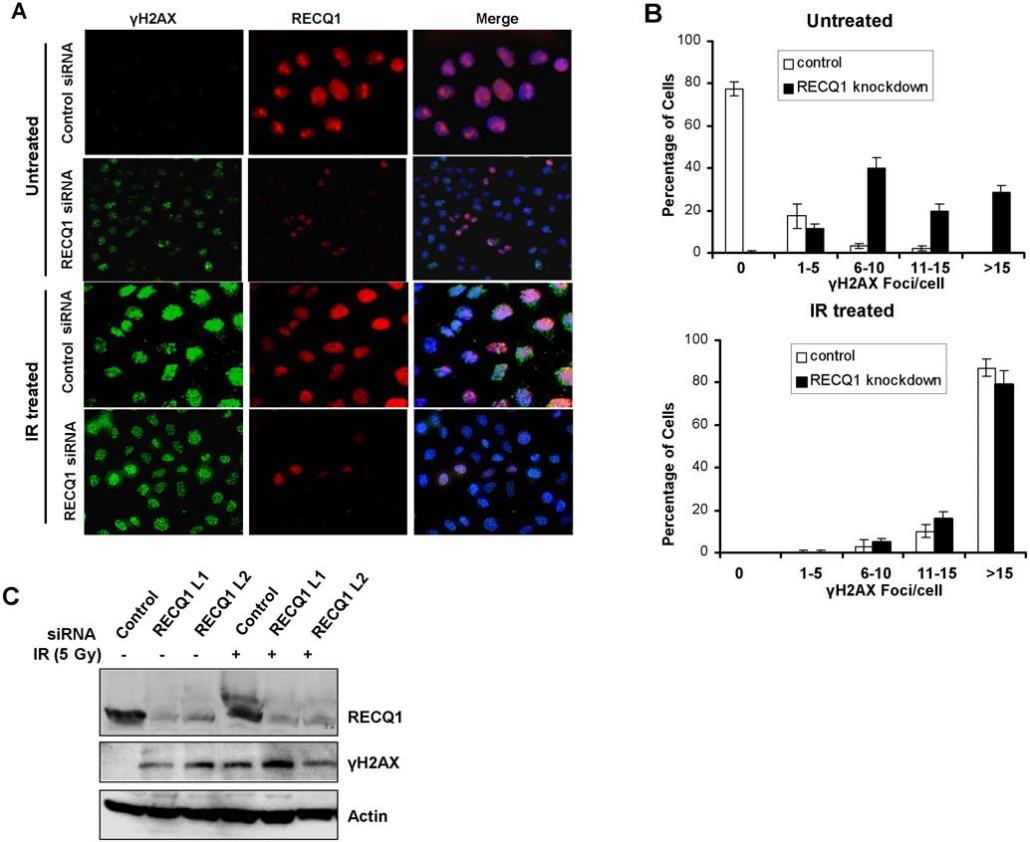
Depletion of RECQ1 leads to spontaneous formation of γ-H2AX foci in the absence of exogenous damage. *Panel A,* Control or RECQ1 siRNA-treated HeLa cells were grown on coverslips, fixed with formaldehyde and co-immunostained with anti- γ-H2AX and anti-RECQ1 antibodies. The merged picture shows cells stained with anti- γ-H2AX (green) and anti-RECQ1 (red) as well as DAPI (blue). Normal induction of γ-H2AX foci is shown upon IR (5 Gy) exposure in RECQ1 depleted cells. *Panel B,* Quantitative assessment of γ-H2AX foci in control or RECQ1-depleted cells that have been either untreated or exposed to IR, as described in *Panel A*. Images of at least 100 cells were captured and used for quantitative analyses of γ-H2AX foci. To avoid bias in the selection of cells, DAPI stained nuclei were randomly selected for γ-H2AX staining. *Panel C,* HeLa cells were treated with control or RECQ1 siRNA. Following IR exposure or not, cell lysates were immunoblotted with anti-RECQ1, anti- γ-H2AX, or anti-actin antibodies.

RecQ helicases are thought to be involved in the maintenance and stabilization of replication forks in response to endogenous stress or exogenous DNA damage [2]. A failure to stabilize forks can lead to fork collapse and double strand breaks. To examine the role of RECQ1 in preventing and responding to double strand breaks, we analyzed γ-H2AX foci formation in control or RECQ1 siRNA transfected cells that were either untreated or exposed to IR (5 Gy). IR exposure resulted in γ-H2AX foci formation; however, in the absence of exogenous DNA damage treatment we observed a significantly greater percentage of RECQ1-depleted cells with an increased number (>5) of γ-H2AX foci compared to control cells (Fig. 8A, 8B). This increased level of γ-H2AX that occurred spontaneously in the RECQ1-depleted cells was also evident by Western blot analysis (Fig. 8C, lane 1 versus lanes 2 and 3 of middle panel).

### RECQ1 depletion leads to increased sister chromatid exchange

**Figure 9:**
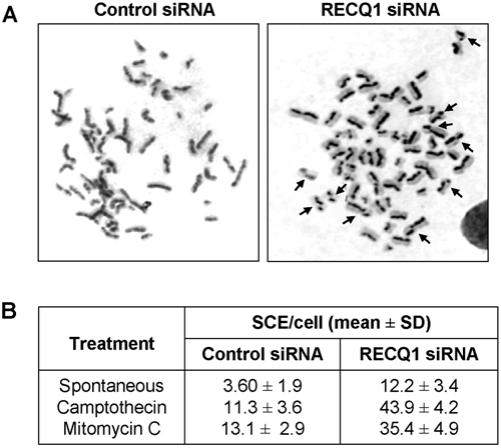
Elevated sister chromatid exchanges in RECQ1-depleted human cells. *Panel A,* SCEs were assayed in BrdU labeled, giemsa-stained chromosome spreads from control or RECQ1 siRNA treated HeLa cells. A representative spread is shown for spontaneous SCEs in control and RECQ1-depleted cells. *Panel B*, Quantitative representation of the number of SCEs per metaphase, either spontaneous or induced by CPT or MMC in cells treated with either control or RECQ1 siRNA. A minimum of 25 metaphases were counted for each cell type and treatment.

The elevated levels of spontaneous γ-H2AX foci in RECQ1-depleted cells may be a consequence of aberrant recombination at sites of double strand breaks. A phenotype associated with defective HR is elevated SCE [1]. Metaphase chromosome preparations from BrdU-labeled RECQ1-siRNA cells or control siRNA cells were differentially stained for detection of SCEs (Fig. 9A). RECQ1-depleted cells displayed a 3.4-fold greater frequency of spontaneous SCEs compared to the control HeLa cells. The fold increase in SCE in the RECQ1-depeleted cells is similar to that reported for HeLa cells depleted of BLM protein or its binding partner BLAP75 [37]. Increased levels of SCE were also observed in the RECQ1-depleted cells that were exposed to the DNA cross-linking agent mitomycin C, as previously published [38], or the topoisomerase inhibitor CPT (Fig. 9B). Importantly, the elevated SCE and γ-H2AX foci suggest that RECQ1 is involved in the resolution of HR intermediates and its absence leads to accumulation of DNA damage.

### RECQ1 interacts with the strand exchange protein Rad51

**Figure 10:**
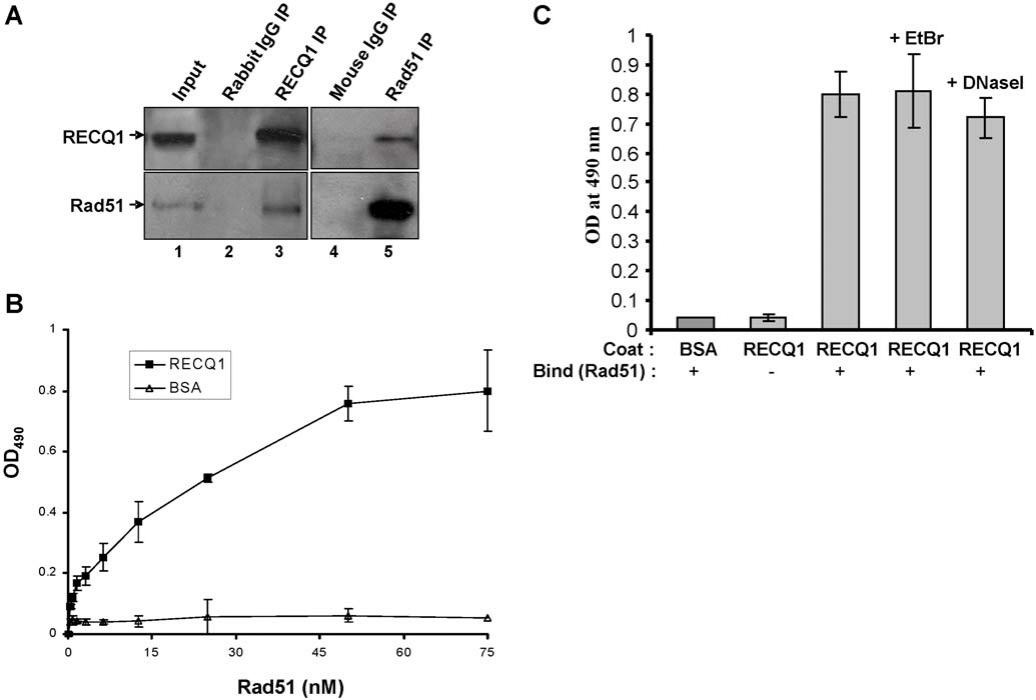
RECQ1 interacts with Rad51. *Panel A,* Co-immunoprecipitation of RECQ1 and Rad51 from HeLa nuclear extracts. Rad51 was detected in RECQ1 immunoprecipitates (lane 3). RECQ1 was immmunoprecipitated with Rad51 from nuclear extracts as detected by Western blot (lane 5). Lanes 2 and 4 represent immunoprecipitate from control rabbit and mouse IgG, respectively. Input (lane 1) represent 10% of the total protein used for immunoprecipitation. *Panels B and C*, ELISA for RECQ1-Rad51 interaction. Either BSA or purified recombinant human RECQ1 (14 nM) was coated onto microtiter plates. Following blocking with 3% BSA, appropriate wells were incubated with the indicated concentrations of purified recombinant human Rad51 (0–76 nM, *Panel B* or 76 nM, *Panel C*) for 1 h at 30°C. In *Panel C* as indicated, DNaseI (100 U/ml) or ethidium bromide (EtBr) (50 µg/ml) was included in the incubation with Rad51 in the binding step in the corresponding wells to test for DNA-mediated protein interaction. Following washing, RECQ1 bound Rad51 was detected by ELISA using rabbit polyclonal antibody against Rad51. The values represent the mean of three independent experiments performed in duplicate with SD indicated by error bars.

A key protein in the strand exchange reaction of HR is Rad51, which facilitates strand invasion of ssDNA into a homologous duplex. To evaluate if Rad51 recombinase and RECQ1 helicase are associated with each other, we performed co-immunoprecipitation experiments using antibodies directed against either Rad51 or RECQ1 to immunoprecipitate the target protein and its potential protein partner from nuclear extracts. The results demonstrate that RECQ1 and Rad51 can be reciprocally immunoprecipitated with each other (Fig. 10A). RECQ1 immunoprecipitate also contained MSH2/6 and MLH1 (data not shown), previously shown to interact with RECQ1 [13]. A direct interaction between Rad51 and RECQ1 was confirmed by ELISA using purified recombinant proteins. Rad51 bound RECQ1 in a protein concentration-dependent manner (Fig. 10B). In control experiments, a very low OD_490_ signal was detected when BSA (0–76 nM) was substituted for RECQ1 or Rad51 was omitted from the binding incubation (Fig. 10B, 10C). The specific binding of Rad51 to the RECQ1-coated wells was analyzed according to Scatchard binding theory as previously described [39], and the apparent dissociation constant (*K_d_*) was determined to be 11.9±2.1 nM. Furthermore, the interaction of RECQ1 and Rad51 was demonstrated to be DNA independent, as evidenced by the similar colorimetric signal observed for RECQ1/Rad51 interaction in the presence of ethidium bromide (EtBr) or DNaseI (Fig. 10C).

## Discussion

Given the genetic linkage of other human RecQ helicases (WRN, BLM, RECQ4) to diseases characterized by premature aging, cancer, and chromosomal instability, we investigated the significance of human RECQ1 for genome integrity, and more specifically its role in the DNA damage response. In this work, we have demonstrated that endogenous human RECQ1 becomes phosphorylated and re-localizes its sub-nuclear distribution to the chromatin fraction upon cellular exposure to DNA damage. Depletion of RECQ1 renders cells sensitive to IR or the topoisomerase inhibitor CPT, and results in spontaneous γ-H2AX foci and elevated SCE, indicating an accumulation of double strand breaks. The biological results suggest that RECQ1 either serves to prevent double strand breaks from forming or directly helps to repair double strand breaks through its interaction with HR repair proteins such as Rad51. Collectively, these studies provide the first evidence for a role of human RECQ1 in the response to DNA damage and chromosomal stability maintenance and point to the vital importance of RECQ1 in genome homeostasis.

The significant reduction in cell proliferation due to RECQ1 depletion in human cells is different from that observed in mice in which RECQ1 deficiency had no obvious effect on the growth/proliferation of mouse embryonic fibroblasts nor the normal development or postnatal growth of mice [8]. The phenotypes of complete loss of human RECQ1 are likely to be more severe than those observed, particularly in the case of shRNA selection, since that protocol presumably selects for clones that fail to completely silence RECQ1. This allows the possibility that RECQ1 is essential for cell viability in humans, in contrast to the case in mice.

It is conceivable that manifestation of cellular or organismal phenotypes in *Recql*-null mice may be masked by other genetic factors. For example, *WRN*-null mice do not exhibit any phenotypes prevalent in WS; however, premature aging phenotypes are observed in a *WRN*-null telomerase-knockout mouse characterized by the presence of critically short telomeres (for review, see [40]). Furthermore, late generation *mTerc-/- WRN -/-* mouse embryonic fibroblasts have an increased load of DNA damage and replicative senescence, properties similar to that observed in human WS fibroblasts.

In addition to their growth defects, RECQ1-depleted human cells display defective maintenance of the G2/M checkpoint following IR exposure and an aberrant apoptotic response to H_2_O_2_-induced stress. The role of RECQ1 in the apoptotic response may be either direct or the effect could be secondary, and the reduction in genotoxic stress-induced apoptosis could be due to the altered cell cycle distribution or viability of the RECQ1-depleted cells.

Intra-nuclear trafficking of nuclear proteins is an important aspect of the DNA damage response since DNA repair factors are known to localize to DNA damage foci. RECQ1 is predominantly found in the nucleoli of proliferating human cells and re-localizes to the nucleoplasm in response to IR-induced DNA damage or replicational stress induced by hydroxyurea. Like RECQ1, WRN and BLM also re-localize to the nucleoplasm after DNA damage [41]–[43]; however, RECQ4 is constitutively present in nuclear foci irrespective of DNA damage [44], suggesting some differences in the sub-cellular distribution of human RecQ helicases before and after DNA damage.

Most of the RECQ1 is loosely tethered to the nucleus; however, a subset of RECQ1 molecules becomes resistant to detergent extraction after IR exposure, probably due to their tighter association with sites of damage in chromatin. Endogenous RECQ1 is phosphorylated in response to DNA damage or replicational stress, and preferentially associates with chromatin. A number of predicted phosphorylation sites for various stress-activated kinases exist in RECQ1. Our unpublished results indicate that RECQ1 is phosphorylated after DNA damage in AT mutated cells, suggesting that ATM is not the major candidate kinase. *In vitro* phosphorylation of RECQ1 by Protein Kinase C or Casein Kinase II modulates its catalytic activities (data not shown). RECQ1 and its protein partners may be localized to sites of DNA damage where RECQ1 utilizes its catalytic activities to process genomic DNA structures as a component of the DNA damage response.

In agreement with this, RECQ1 deficiency in both mouse [8] and human cells resulted in an increased sensitivity to IR or CPT which introduce strand breaks that can be converted to double strand breaks during replication. Spontaneously elevated γ-H2AX foci and SCEs observed in RECQ1-depleted cells (this study and reference [38]) may be due to unsuccessful attempts to ‘repair’ damaged replication forks by HR at double strand breaks. Thus, RECQ1 may have a direct role in repairing double strand breaks. Alternatively, RECQ1 may act to prevent double strand breaks from occurring in the first place. The latter explanation would be consistent with our observation that only a minority of RECQ1 co-localizes with γ−H2AX foci. The sensitivity of RECQ1 deficient cells to other DNA damaging agents remains unexplored and RECQ1 might also participate in other DNA repair pathways.

Strong candidates for RECQ1 protein interactors that serve to suppress cross-over of sister chromatids are recombination proteins, mismatch repair factors that regulate genetic recombination [13], and Type IA topoisomerases that have been implicated genetically and biochemically to collaborate with RecQ helicases to preserve chromosomal integrity [2]. It is conceivable that Rad51 may act to regulate RECQ1 helicase activity on HR intermediates (D-loop, Holliday Junction) that RECQ1 has the ability to unwind [10] since Rad51 plays a critical role in the strand invasion step of HR repair of strand breaks. However, we did not detect an effect of Rad51, inhibitory or stimulatory, on RECQ1 catalyzed unwinding of a variety of oligonucleotide-based DNA substrates including forked duplex, D-loop, or synthetic Holliday Junction (data not shown). RECQ1 may utilize its motor ATPase function to displace Rad51 from inappropriate strand invasion intermediates that are either homeologous or occur in an untimely manner. A similar function was proposed for the yeast Srs2 helicase which physically binds to Rad51 [45] and displaces the strand exchange protein from DNA [45], [46].

Our observation that human RECQ1 directly interacts with Rad51 is consistent with other reports that RecQ helicases (BLM, Sgs1 [47]) function in DNA repair through HR by their physical interactions with Rad51. It was proposed that the physical interaction between BLM and Rad51 serves to recruit BLM to sites of recombinational repair and possibly load BLM onto Holliday Junctions in a particular orientation that would then dictate the direction of junction translocation. The spontaneously elevated nuclear Rad51 foci observed in BS cells [47] and *Recql* ^−/−^ cells [8] may represent accumulation of unresolved recombination intermediates or a greater load of endogenous DNA damage in the absence of the RecQ helicase. Our earlier demonstration that RECQ1 interacts physically and functionally with mismatch repair proteins [13] is likely to be important in the mechanisms whereby RECQ1 regulates genetic recombination. MSH2/6 proteins may interact with RECQ1 to unwind recombination intermediates that contain mismatches, as proposed for the mechanism of Sgs1 to prevent homeologous recombination [48].

It was suggested that the fission yeast Rqh1 helicase functions after Rad51 focus formation during DNA repair through its interaction with Topoisomerase III in G2 [49]; however, Rqh1 and other RecQ helicases may have early and late roles in homologous recombination [50]. Human RECQ1 was reported to physically interact with Topo IIIα [14], and Rad51 focus formation after DNA damage is not dependent on RECQ1 in mouse cells [8]. It is likely that RECQ1 utilizes its catalytic helicase and strand annealing activities to facilitate the resolution of HR repair intermediates with other proteins intimately involved in this process. The observed elevated SCE after CPT-induced DNA damage is consistent with this proposal.

BLM has been proposed to suppress SCEs by its concerted action with Top3α [51]; however, RECQ1, WRN, and RECQ5β were not able to substitute for BLM in Top3α mediated double Holliday Junction dissolution on model DNA substrates *in vitro* [52]. *RECQ1* ^−/−^ *BLM* ^−/−^ or *RECQ5* ^−/−^ *BLM* ^−/−^ DT40 cells displayed a growth defect, and *RECQ5* ^−/−^ *BLM* ^−/−^ cells had a higher SCE frequency compared with *BLM* ^−/−^ cells [6]. An independent role of RECQ5 in sister chromatid exchange suppression was demonstrated in embryonic stem cells or differentiated fibroblasts from *RECQ5*-knockout mice [7]. Primary embryonic fibroblasts from the RECQ1 knockout mouse display spontaneously elevated SCEs and chromosomal aberrations [8], suggesting that RecQ helicases participate in non-redundant pathways to suppress cross-overs during mitosis.

Although a genetic disorder has not yet been linked to a mutation in RECQ1, recent analyses of *RECQ1* single nucleotide polymorphisms (SNPs) have identified an association of RECQ1 with a reduced survival of pancreatic cancer patients [53], [54]. RECQ1 SNPs displayed significant genetic interaction with SNPs in the homologous recombinational repair genes ATM, RAD54L, XRCC2 and XRCC3. A role of RECQ1 in HR is further suggested by the observation that SNPs in RECQ1 affect the response to the anti-cancer drug gemcitabine induced radio-sensitization that selectively requires functional HR [53], [54]. The chromosomal instability arising from RECQ1 deficiency may contribute to a cancer predisposition. RECQ1 is differentially up-regulated in transformed cells or cells that are actively proliferating [5]. We propose that RECQ1 confers genomic integrity in transformed or actively proliferating cells.

The human *RECQ1* gene is localized to chromosome 12p11-12 [3], [55], a location of instability in testicular germ-cell tumors [56]. *RECQ1* expression in mouse is highest in the testis [8]. The purification of a RECQ1 ribonucleoprotein complex from rat testis with testis-specific small non-coding piRNAs involved in gene silencing, cell growth, and development [57] raises the question of what is the precise mechanism whereby RECQ1 and its associated piRNA complex regulates the germ-line. The mechanistic similarities between piRNA synthesis and DNA replication [58] suggest that RECQ1 and its associated DNA replication/repair proteins (RPA, EXO-1, MSH2/6) are involved in piRNA biogenesis. This is an unexpected and potentially exciting role for RECQ1 protein in the metabolism of regulatory small RNAs important for development and mechanisms of inheritance. Presently, it can only be speculated if the piRNA complex regulates the genome at the level of DNA or histones, or at a posttranscriptional level. Interestingly, QDE-3, the RECQ1 homolog in *Neurospora crassa*, has also been implicated in gene silencing [59]. The *qde-3* mutant was found to be hypersensitive to a variety of DNA mutagens and exhibit increased mutability and extensive chromosomal deletions [60]–[62], implicating a broader role of the *Neurospora* RECQ1 homolog in the DNA damage response and chromosomal stability. Our cellular studies implicate a unique and important role of human RECQ1 in genome homeostasis as well. Further work is required to understand RECQ1-piRNA genome defense association. Clearly, RECQ1 has become the latest member of the RecQ helicase family with important biological functions through its involvement in the DNA damage response and fundamental processes of chromosomal nucleic acid metabolism.

## Supporting Information

Figure S1 Supplementary Data Figure
(25.27 MB TIF)
