# Supplementary figures and images for: Human RECQ1 Is a DNA Damage Responsive Protein Required for Genotoxic Stress Resistance and Suppression of Sister Chromatid Exchanges
Source: PLoS One. 2007 Dec 12;2(12):e1297. doi: 10.1371/journal.pone.0001297 (PMC2111050; doi:10.1371/journal.pone.0001297)

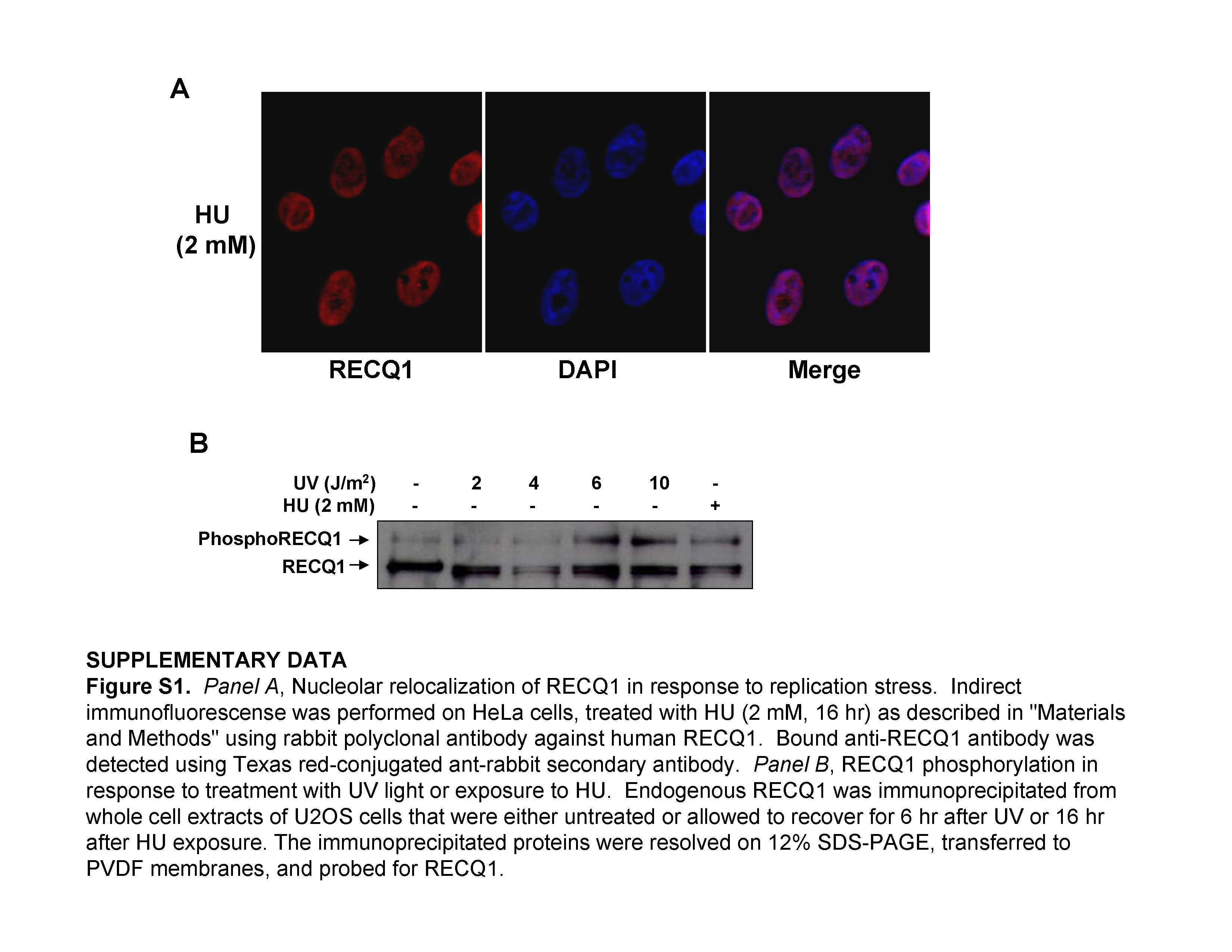

Supplement: Figure S1 — Supplementary Data Figure (25.27 MB TIF) [file pone.0001297.s001.tif]
